# Early Vaginal Incision in Septic Pelvic Disease

**Published:** 1897-10-30

**Authors:** 


### EARLY VAGINAL INCISION IN SEPTIC PELVIC DISEASE.

Some of the most interesting of the papers read at the Montreal meeting of the British Medical Association were those contributed by Americans, and detailing American methods and experiences. Among these was a careful appreciation of the various operative procedures at our disposal in dealing with septic pelvic disease, by Dr. Fernand Henrotin[1], of Chicago, who strongly urged that recourse should be had in many such cases to an early vaginal incision. The paper is not one which lends itself to condensation, but we draw attention to it not only because it shows that recourse can usefully be had to the vaginal route in many cases at a stage when laparotomy is out of the question, but also because it illustrates the reasons that have led to the much wider acceptance of this route of access to pelvic disease in recent years. There is very little doubt that the cause of the disrepute into which the vaginal route fell in the early days of gynæcology, was due to the use of punctures and niggling incisions, and to inefficient methods of drainage. Free incisions and the use of iodoform gauze as a means of drainage have quite altered the aspect of many questions. It must be distinctly borne in mind that when Dr. Henrotin advocates "early vaginal incision," what he has in his mind is not a puncture in the dark, but a very free exploration, opening up the cavity of the peritoneum, followed by effectual drainage.

[1] Brit. Med. Jour., Oct. 23rd.

